# Development of a Pericapsular Knee Desensitization Technique in Dogs: An Anatomical Cadaveric Study

**DOI:** 10.3390/vetsci12060599

**Published:** 2025-06-19

**Authors:** Marta Garbin, Raiane A. Moura, Yasmim C. Souza, Mariana Cavalcanti, Adam W. Stern, Marta Romano, Enzo Vettorato, Pablo E. Otero, Diego A. Portela

**Affiliations:** 1 Department of Clinical Science, Faculté de Médecine Vétérinaire, Université de Montréal, Saint-Hyacinthe, QC J2S 8H5, Canada; marta.garbin@umontreal.ca; 2 Department of Comparative, Diagnostic, and Population Medicine, College of Veterinary Medicine, University of Florida, Gainesville, FL 32608, USA; 3 Department of Anesthesiology and Pain Management, Facultad de Ciencias Veterinarias, Universidad de Buenos Aires, Buenos Aires C1427CWO, Argentina

**Keywords:** analgesia, chronic pain, dog, interventional pain medicine, joint, osteoarthritis pain, regional anesthesia, stifle, ultrasound

## Abstract

Regional anesthesia techniques targeting articular nerve branches offer promising avenues for managing articular pain. This study developed and compared the success rates of an ultrasound-guided versus a blind pericapsular knee desensitization (PKD) technique in canine cadavers. In Phase I, gross dissection and ultrasound evaluations were performed in eight limbs to characterize the anatomy of the medial (MAN), lateral (LAN), and posterior (PAN) articular branches of the saphenous, common fibular, and tibial nerves, respectively, and to identify suitable anatomical and ultrasonographic landmarks. In Phase II, ultrasound-guided and blind PKD injections of a dye solution were randomly performed in 10 cadavers (20 limbs), followed by dissection and histological assessment of staining accuracy. The ultrasound-guided technique achieved a significantly higher overall success rate (96.7%) than the blind technique (73.3%; *p* = 0.02). The MAN was successfully stained in 100% of ultrasound-guided and 50% of blind injections (*p* = 0.03), while the LAN and PAN were stained with high but comparable success. Parent nerve involvement was minimal for MAN and PAN but frequent for the common fibular nerve following LAN injections. Histological confirmation supported the anatomical findings, although PAN identification remained inconsistent. These results support the feasibility and increased precision of ultrasound-guided PKD, providing a foundation for further clinical evaluation.

## Simple Summary

Chronic pain from stifle joint diseases, such as osteoarthritis, is a common condition in dogs that often requires complex medical and surgical management. Conventional analgesic methods may be inadequate for controlling pain or may be associated with undesirable side effects, which may lead to diminished quality of life. Peripheral nerve blocks (i.e., femoral and sciatic nerve blocks) can be employed to relieve joint pain but are associated with unwanted motor impairment. This cadaveric study introduced the pericapsular knee desensitization (PKD) technique as a motor-sparing approach to selectively target the sensory articular branches innervating the canine knee joint. Two techniques—ultrasound-guided and blind injections—were developed and compared to evaluate the success rate of staining the articular nerves using a dye solution. The results showed that the ultrasound-guided technique had significantly greater accuracy in staining the target nerves. These findings support the feasibility of the PKD technique and provide anatomical and procedural guidance for future clinical studies aimed at improving pain management in dogs with stifle disease.

## 1. Introduction

Osteoarthritis is a progressive joint disorder characterized by chronic pain, a gradual decline in limb function, and diminished quality of life [1]. In dogs, osteoarthritis is highly prevalent, affecting approximately 40% of young individuals (i.e., eight months to four years) and over 50% of elderly dogs (i.e., >eight years) [2,3]. Managing canine osteoarthritis commonly involves a multimodal approach that includes pharmacological agents, nutritional strategies, and rehabilitation therapies [4,5].

In human medicine, therapeutic nerve blocks and radiofrequency ablation of the articular branches supplying the knee joint are widely employed for perioperative analgesia, chronic pain management, and emergency care [6,7,8]. Similarly, therapeutic nerve blocks are showing promise in veterinary medicine for managing chronic pain in dogs [9,10,11]. Moreover, selective pericapsular desensitization approaches have been developed for other canine joints, including the pericapsular hip desensitization (i.e., PHD) [11,12] and the pericapsular elbow desensitization (i.e., PED) [13] techniques.

The canine knee joint capsule is primarily innervated by three articular nerves: the medial articular nerve (MAN), which originates primarily from the saphenous nerve; the posterior articular nerve (PAN), which originates from the tibial nerve; and the lateral articular nerve (LAN), which originates from the common fibular nerve. Occasionally, the obturator nerve also contributes to the MAN [14,15,16]. Although partial stifle desensitization techniques have been described [9,17,18], no study to date has been designed to target the entire sensory innervation of the canine knee joint capsule or to establish an ultrasound-guided pericapsular knee desensitization (PKD) technique.

Therefore, the present cadaveric study aimed to develop a regional anesthesia technique to selectively target the articular nerve branches responsible for the sensory innervation of the entire canine knee joint. Additionally, it evaluated the success rates of articular nerve staining using either a blind or ultrasound-guided approach. The study hypothesized that ultrasound guidance would improve the success rate of articular nerve staining compared to the blind technique.

## 2. Materials and Methods

This prospective, blinded, randomized, anatomical cadaveric study utilized 14 adult mixed-breed canine cadavers (i.e., 28 pelvic limbs) that had either been euthanized or had died due to medical conditions unrelated to the study and were donated by their owners to the University of Florida, Small Animal Veterinary Teaching Hospital for teaching or research purposes. All the cadavers were stored frozen at −80 degrees Celsius until use. According to institutional policy, the University of Florida does not require IACUC protocol approval for research involving cadavers of animals that were euthanized or died for reasons unrelated to the study. Before use, all the cadavers were thawed at room temperature for 36–48 h, and the hair over the pelvic limbs was clipped. Cadavers were excluded if they exhibited any visible abnormalities of the pelvic limbs, surgical incision marks near the stifle, or a body condition score < 2 or >7 out of 9.

The study consisted of two phases. In Phase I, the anatomical relationships between the knee articular nerves and surrounding structures were identified to determine potential landmarks for blind and ultrasound-guided injections. In Phase II, the injectate spread and nerve staining following blind and ultrasound-guided injections of the MAN, PAN, and LAN were evaluated and compared. Furthermore, tissues identified as potential branches of the articular nerves were removed and analyzed histologically.

- Phase I: Gross anatomical dissection and sonoanatomy of the pericapsular region of the knee

The objectives of this phase were (1) to analyze the origin, course, and termination of the major articular nerves as they branch to the canine knee joint capsule and its boundaries; (2) to assess the ultrasonographic anatomy of the pericapsular region; and (3) to develop a PKD technique to target the articular nerves using a blind or an ultrasound-guided approach.

**Figure 1:**
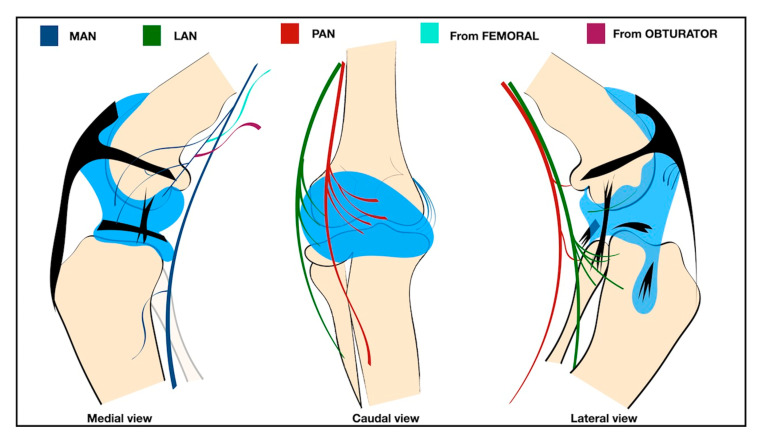
Schematic representation of the sensory innervation of the knee in a dog. MAN: medial articular nerve, a branch of the saphenous nerve (blue); PAN: posterior articular nerve, branches of the tibial nerve (red); LAN: lateral articular nerve, branches of the fibular nerve (green); branches of the femoral nerve (sky blue) and branches of the obturator nerve (purple).

A total of four thawed canine cadavers (i.e., eight pelvic limbs) were utilized in this phase. Based on the previously described anatomy of the knee capsule innervation in dogs [14], the saphenous, tibial, and common fibular nerves were examined to characterize the anatomical features of the MAN, PAN, and LAN, respectively (Figure 1).

Initially, in the first two cadavers, the medial and lateral aspects of the right thigh were dissected to identify the location of the saphenous, tibial, and common fibular nerves and their articular branches entering the knee joint capsule. The skin, fascia, and surrounding musculature were removed to expose relevant anatomical structures. When necessary, a surgical binocular microscope (Storz Urban US-1 surgical microscope; Storz, MO, USA) was used to aid in visualizing articular nerve branches. The anatomical relationships between each parent nerve and the surrounding intermuscular planes or vascular structures were recorded, along with the paths of the articular branches after they branched from the parent nerves. These observations included their proximity to vascular and bony landmarks, aiding in identifying potential landmarks for blind and ultrasound-guided injection techniques.

To establish ultrasonographic landmarks for the ultrasound-guided PKD technique and define potential needle trajectories for targeting the MAN, PAN, and LAN, two experienced anesthesiologists performed an ultrasound evaluation of the contralateral limb of each cadaver using a portable ultrasound machine (SonoSite Edge; SonoSite Inc., Bothell, WA, USA) equipped with a linear transducer (15–6 MHz HFL50× or 13–6 MHz L25×; SonoSite Inc.).

Once the landmarks were established, trial injections of 0.1 mL of a yellow dye solution per nerve were performed in the remaining two cadavers of Phase I, with each cadaver receiving a blind injection in one pelvic limb and an ultrasound-guided injection in the contralateral limb. Following these procedures, all the injected limbs were dissected to assess dye localization around the target nerves and, if necessary, to refine the injection technique for improved accuracy and application.

To enhance ultrasound visualization of vessels adjacent to the knee’s articular nerves, the femoral artery and vein in the proximal femoral triangle were catheterized, proximally ligated, and infused with ultrasound gel, which rendered them distended and hypoechoic. This method delineated the femoral, descending genicular, and popliteal arteries on ultrasound as previously described [19]. The dye solutions were prepared by adding 1 mL permanent yellow or green tissue dye (Davison Marking System; Bradley Products Inc., Bloomington, MN, USA) in 50 mL 0.9% NaCl (Baxter Healthcare Corporation, Deerfield, IL, USA) and injected via a 22-gauge, 38 mm Quincke spinal needle (BD Spinal needle, Becton, Dickinson and Company, Franklin Lakes, NJ, USA) connected to a prefilled syringe.

- Phase II: Comparison of ultrasound-guided versus blind PKD techniques

This phase aimed (1) to compare the success rate of staining the articular branches innervating the knee joint capsule using either a blind or ultrasound-guided PKD technique; (2) to histologically evaluate if the tissues identified as potential branches of the articular nerves were nervous in origin.

A total of 10 thawed cadavers (i.e., 20 pelvic limbs) were utilized in this phase. This number was determined following a priori sample size calculation in which an alpha level of 0.05 and a power of 0.8 were set. By subjectively estimating an overall success rate of 40% for blind injections and 95% for ultrasound-guided injections, a total of 10 pelvic limbs were needed per group. The right and left pelvic limbs of each cadaver were randomly assigned using an online randomization tool [www.random.org/lists/ (accessed on 2 April 2024)] to one of the following groups: ultrasound-guided PKD injections (Group US) and blind PKD injections (Group B). In both groups, the injection sites and needle trajectories targeting the MAN, PAN, and LAN were based on the anatomical and sonographic findings of Phase I. In all cases, a 22-gauge, 38 mm Quincke spinal needle was used to administer 0.025 mL kg^−1^ of a green dye solution prepared as described in Phase I to target the MAN and LAN, and 0.05 mL kg^−1^ to target the PAN.

### 2.1. Ultrasound-Guided PKD Injections

**Figure 2:**
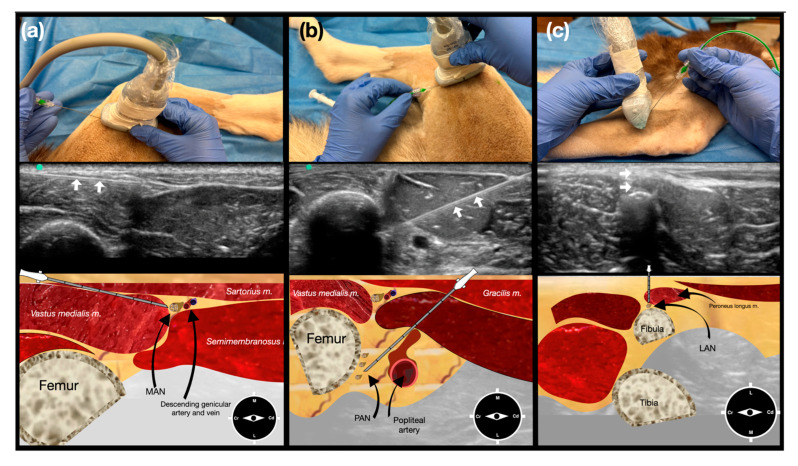
Ultrasound-guided pericapsular knee desensitization (PKD) technique. Column (**a**) Ultrasound-guided medial articular nerve (MAN) injection. The dog is placed in lateral recumbency with the limb to be injected downwards. The transducer is placed transversely over the distal third of the femur, with the marker oriented cranially. The needle is introduced in-plane from the cranial aspect of the transducer. The ultrasound image shows the medial aspect of the region, the needle trajectory (white arrows), and a schematic representation of the target injection fascial plane. Column (**b**) Ultrasound-guided posterior articular nerve (PAN) injection. The dog is placed in lateral recumbency with the limb to be injected downwards. The transducer is placed transversely over the distal third of the femur, with the marker oriented cranially. The needle is introduced in-plane from the caudal aspect of the transducer. The ultrasound image shows the popliteal fossa, the needle trajectory (white arrows), and a schematic representation of the target injection site. Column (**c**) Ultrasound-guided lateral articular nerve (LAN) injection. The dog is placed in lateral recumbency with the limb to be injected uppermost. The transducer is placed transversely over the distal third of the femur, with the marker oriented cranially. The needle is introduced out-of-plane and advanced through the belly of the fibularis longus to the caudolateral aspect of the fibular head. The ultrasound image shows the fibula, the needle trajectory (white arrows), and a schematic representation of the target injection site.

For the MAN approach, the transducer was placed transversely over the distal third of the femur, with the marker oriented cranially, to obtain a short-axis view of the femur and identify the vastus medialis, semimembranosus, and the sartorius muscles. An in-plane craniocaudal needle approach was then used to deposit dye into the fascial plane between these muscles, in correspondence with the descending genicular artery, where the MAN is located (Figure 2a).

For the PAN approach, the transducer was positioned on the medial aspect of the distal thigh to identify the gastrocnemius muscle and femoral artery. Once the femoral artery was visualized in the short axis, the transducer was slid distally until the bifurcation of the femoral artery into the popliteal and distal caudal femoral arteries was identified. The popliteal artery was then followed distally to its location within the intercondylar fossa. An in-plane caudocranial needle approach was used to deposit the dye adjacent to the cranial aspect of the popliteal artery (Figure 2b).

For the LAN approach, the transducer was oriented perpendicular to the tibial long axis and placed above the lateral tibial condyle, with the marker directed cranially. In this position, the fibular head and the fibularis longus muscle were identified. After centering the fibular head on the ultrasound screen, the needle was introduced out-of-plane and advanced through the belly of the fibularis longus to the caudolateral aspect of the fibular head, where the dye solution was deposited when the needle tip contacted the periosteum of the head of the fibula (Figure 2c).

### 2.2. Blind PKD Injections

**Figure 3:**
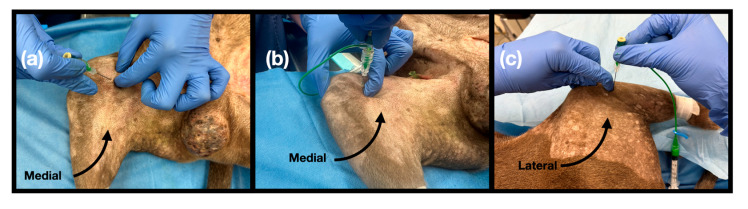
Injection points for blind injections targeting the articular branches. (**a**) Medial articular nerve (MAN) injection. The dog is placed in lateral recumbency with the limb to be injected downwards. The injection site is located by palpating the depression formed between the vastus medialis and semimembranosus muscles on the medial aspect of the thigh, proximal to the knee joint. The needle is inserted perpendicular to the skin and advanced slightly into the subcutaneous tissues, aiming to inject the dye solution deeper to the sartorius muscle (**b**) Posterior articular nerve (PAN) injection. The popliteal fossa is palpated to identify the depression between the medial and lateral bellies of the gastrocnemius muscle. The needle is inserted perpendicular to the skin at the caudal aspect of the knee joint and advanced to a depth of 1–1.5 cm. (**c**) Lateral articular nerve (LAN) injection. The caudolateral margin of the fibular head serves as the primary landmark. The needle is introduced perpendicular to the skin until it contacts the caudolateral aspect of the fibular head.

For the MAN approach, the injection site was located by palpating the depression formed between the vastus medialis and semimembranosus muscles on the medial aspect of the thigh, proximal to the knee joint. A subtle but consistent depression was palpable in this region. The needle was inserted perpendicular to the skin and advanced slightly into the subcutaneous tissues, aiming to inject the dye solution deeper into the sartorius muscle, within the fascial plane containing the MAN (Figure 3a).

For the PAN approach, the popliteal fossa was palpated to identify the depression between the medial and lateral bellies of the gastrocnemius muscle. The needle was inserted perpendicular to the skin at the caudal aspect of the knee joint and advanced to a depth of 1–1.5 cm, aiming for the vicinity of the popliteal artery where the PAN is located (Figure 3b).

For the LAN approach, the caudolateral margin of the fibular head served as the primary landmark. The needle was introduced perpendicular to the skin until it contacted the caudolateral aspect of the fibular head, targeting the expected course of the LAN within this region (Figure 3c).

### 2.3. Gross Anatomical Dissection

After all the injections were completed, each limb was immediately dissected by two unblinded investigators. Nerve staining was evaluated by a third blinded investigator and documented as successful when the target nerve was stained around its entire circumference for both the MAN and LAN and when the tissues between the popliteal artery and the external surface of the caudo-proximal quadrant of the knee capsule were stained for the LAN. Additionally, staining of the external surface of the joint capsule was evaluated in the lateral, medial, and caudal quadrants. Dye spread toward the parent nerve and the presence of the dye solution in unwanted locations (e.g., intravascular or intracapsular) was also assessed and noted.

The stained tissues of the injection sites, presumably containing the MAN, PAN, and LAN, were surgically removed. The tissues were fixed in 10% neutral buffered formalin and processed for histological examination. For analysis, the tissues were embedded in paraffin, sliced into 4 µm sections, and stained using hematoxylin and eosin. The primary objective of the histological assessment was to identify whether nerve tissue was present within the collected samples. Histological sections were evaluated by a board-certified pathologist blinded to the technique allocation

### 2.4. Statistical Analysis

The overall proportion of nerves stained with the ultrasound-guided or blind technique (i.e., success rate) was calculated by dividing the number of stained nerves by the number of target nerves per limb. A two-sided Fisher’s exact test was used to compare the proportion of the overall nerve staining and the proportion of each target nerve staining with the two techniques. The categorical data are presented as percentages [95% confidence interval (CI)] and the continuous data as the median and 25th and 75th percentiles (Q1–Q3). Differences were considered significant when *p* < 0.05. Statistical analyses were performed using GraphPad Prism Version 8.0 (GraphPad Software Inc., San Diego, CA, USA).

## 3. Results

- Phase I: Anatomical findings

At the mid-thigh level, the MAN was observed originating from the saphenous nerve and traveling cranially toward the medial aspect of the knee joint capsule. It coursed within the fascial plane between the vastus medialis and semimembranosus muscles, deeper to the separation between the cranial and caudal bellies of the sartorius muscle, accompanied by the descending genicular artery, which branches directly from the femoral artery near the origin of the saphenous artery. Near the medial epicondyle of the femur, the MAN divided into several smaller branches before reaching the knee joint capsule.

Multiple small nerve branches were observed emerging from the tibial nerve and coursing cranially toward the caudal aspect of the knee joint capsule. However, the complete trajectory of the PAN and its terminal branches could not be distinctly isolated or traced, likely due to the abundant surrounding connective tissue.

The common fibular nerve was observed coursing superficially to the lateral digital extensor muscle. Along its trajectory, it gave rise to several small articular branches (i.e., LAN). These branches traveled between the cranial margin of the lateral digital extensor muscle and the caudal margin of the fibularis longus muscle, ultimately terminating near the head and neck of the fibula and the lateral aspect of the knee joint capsule.

Ultrasonography and gross anatomical landmarks allowed for the consistent identification of key anatomical landmarks considered relevant for injection (Figure 2 and Figure 3). The interfascial plane between the vastus medialis and semimembranosus muscles, deep to the sartorius muscle, was proposed for the MAN approach, the popliteal artery was proposed for the PAN approach, and the fibular head was proposed for the LAN approach. Accordingly, an in-plane needle approach was used for the MAN and PAN, while an out-of-plane approach was used for the LAN.

- Phase II: Comparison of ultrasound-guided versus blind PKD techniques

The ten canine cadavers used in this phase were four males and six females, with a body mass of 15.9 (8.5–28.1) kg and a body condition score of 4 (2.7–5) out of 9.

**Figure 4:**
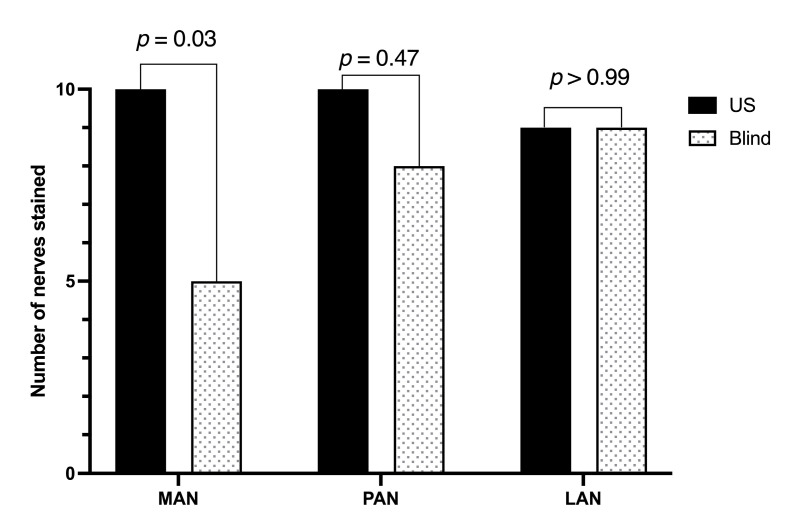
Number of knee articular nerve branches stained in 10 canine cadavers (20 pelvic limbs) using ultrasound-guided (US) and blind pericapsular knee desensitization (PKD) techniques. Each technique was applied to 10 limbs.

A total of 30 injections were performed per technique, targeting the MAN, PAN, and LAN (*n* = 10 each). The overall success rate was 29/30 [96.7% (95% CI 99–86%)] in the group US and 22/30 [73.3% (95% CI 85–55%)] in the group B (*p* = 0.02). The MAN was successfully stained in 100% of ultrasound-guided injections and 50% of blind injections (*p* = 0.03). The PAN was stained in 100% of ultrasound-guided injections and 80% of blind injections, while the LAN was successfully stained in 90% of injections with both techniques (Figure 4).

None of the saphenous nerves were stained following MAN injection with either technique. The tibial nerve was stained in only one case (10%) after PAN injection with the ultrasound-guided technique (*p* = 0.1). After LAN injections, the common fibular nerve was stained in 9 cases (90%) of group US and in 8 cases (80%) of group B (*p* = 1).

In both groups, US and B, intravascular dye contamination of the descending genicular artery following MAN injection was found in one case. Additionally, in group US, popliteal artery contamination was found in two cases, while intra-articular contamination following PAN injection was detected in two cases in group B.

No staining was observed in the lateral or medial quadrants of the joint capsule across all cases. Instead, staining of the caudal quadrant of the capsule was achieved in 100% of extracapsular injections targeting the PAN in both groups.

**Figure 5:**
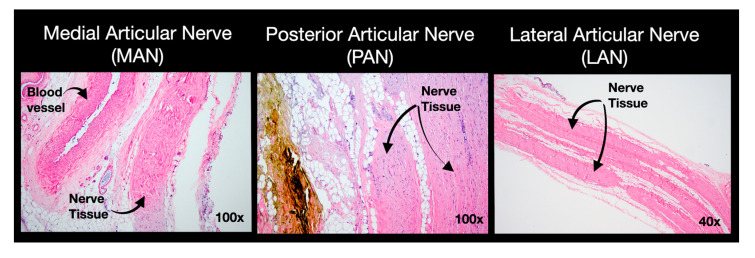
Histological sections of sampled tissues following pericapsular knee desensitization (PKD) injections in mixed-breed canine cadavers. The microscopic images at 40× and 100× magnification revealed nerve fascicles from the targeted medial, posterior, and lateral articular nerves. Sections were stained with hematoxylin–eosin; photomicrographs were obtained using a digital camera (DP74, Olympus, Redmond, WA, USA) attached to a microscope (BX43, Olympus, Redmond, WA, USA) and imaging software (cellSens, Version 4.2, Olympus, Redmond, WA, USA).

Histological analysis revealed that the MAN and LAN were present in 95% of the samples submitted for each limb, while the PAN was present in 55% of the submitted samples. Articular nerves were found in 76.7% (95% CI 88–59%) and 86.7% (95% CI 94–70%) of the histological samples for groups US and B, respectively (*p* = 0.50). Histological analysis revealed that identifying the PAN branches remained inconsistent (Figure 5).

## 4. Discussion

The present study identified three reliable injection points to selectively target articular nerve branches responsible for sensory innervation of the canine knee joint capsule and demonstrated the feasibility of blind and ultrasound-guided PKD techniques. The results showed that the ultrasound-guided PKD technique yielded a higher overall success rate in staining the articular nerves compared to the blind technique. Specifically, ultrasound guidance significantly improved the accuracy of staining the MAN, emphasizing the benefit of using ultrasound guidance for real-time needle tip and landmark visualization for precise injectate placement. While no significant difference in staining success was observed between the two techniques for the LAN and PAN, a high success rate (i.e., >90%) was maintained for both nerves under ultrasound guidance. This lack of a significant difference for LAN and PAN may arise from a limited sample size for individual nerve analysis or indicate a need for further refinement of the technique to enhance accuracy for these specific approaches targeting these nerve branches. In dogs, the MAN primarily innervates the craniomedial and caudal knee joint regions, encompassing the medial collateral ligament, infrapatellar fat pad, and attachments of the cruciate ligaments and meniscal horns. In contrast, the PAN selectively targets mainly the caudal joint capsule, while the LAN supplies the lateral/caudolateral capsule, lateral collateral ligament, lateral meniscus, and superior tibiofibular joint [14,15,16]. The MAN’s extensive distribution to superficial and intra-articular structures, particularly its branches to the cruciate ligaments and meniscal horns, underscores its critical role in nociceptive signaling in the knee. These anatomical findings support the clinical rationale for ultrasound-guided pericapsular techniques targeting the three main components of the innervation of the knee joint capsule in dogs.

In human medicine, periarticular sensory blocks of the knee are commonly referred to as genicular nerve blocks. These techniques, initially developed for chronic pain management and subsequently adapted for perioperative analgesia, target articular branches of the femoral, saphenous, tibial, and common fibular nerves [7,20]. Ultrasound-guided adaptations have enhanced precision and enabled quadriceps-sparing analgesia, offering an alternative to femoral or adductor canal blocks [20]. Genicular nerve blocks and radiofrequency ablation have been shown to be reliable alternative techniques to intra-articular corticosteroids or systemic analgesics for providing analgesia for knee osteoarthritis in humans [21,22,23]. In translating these interventional strategies to veterinary medicine, Boesch et al. [24] demonstrated the feasibility of radiofrequency ablation in dogs by inducing controlled degeneration of the saphenous nerve, providing promising preliminary evidence for advancing regional anesthesia techniques as part of interventional procedures.

The saphenous nerve block has been used as an alternative to the femoral nerve block for providing analgesia in stifle surgery in dogs without inducing motor impairment of the quadriceps muscle [25]. However, O’Connor and Woodbury [14] reported that branches of the obturator (2/18 specimens; 11%) or femoral nerves (2/18 specimens; 11%) merged with the MAN after its origin from the saphenous nerve, contributing to the innervation of the knee capsule. Recently, Di Franco et al. [26] reported improved analgesic outcomes in dogs undergoing stifle surgery when an obturator nerve block was added to saphenous and sciatic nerve blocks, suggesting that obturator nerve desensitization may contribute to more effective stifle joint analgesia. The ultrasound-guided MAN approach described in this study directly targets the MAN before its final ramification, thus carrying the potential advantage of desensitizing not only the branches originating from the saphenous nerve but also the branches from the obturator and femoral nerves, which could be missed with the traditional saphenous nerve block approach [17].

Gingold et al. [18] described a motor-sparing regional technique for stifle surgery in dogs, aiming to desensitize articular nerve branches of the tibial nerve via ultrasound-guided infiltration around genicular structures (i.e., FLAGS block). Inspired by the human interspace between the popliteal artery and the posterior capsule of the knee (IPACK) block [27], this approach targets the popliteal fossa by positioning the needle between the femur and joint capsule cranially and the popliteal artery caudally, delivering local anesthetic to block the sensory joint branches of the tibial nerve (i.e., the PAN). While Gingold et al. [18] based their approach on anatomical reasoning and clinical observations, the present study provided direct anatomical evidence supporting this strategy. By confirming consistent staining of the PAN in a cadaveric model, our findings strengthen the anatomical rationale behind the FLAGS technique and offer additional support for its clinical use. While the FLAGS block focuses solely on the PAN, this study evaluates a more comprehensive approach by targeting all the major articular nerves of the knee joint capsule (i.e., MAN, PAN, and PLAN).

The proximity of the common fibular nerve to the LAN injection site resulted in its inadvertent staining in several specimens, suggesting a potential inadvertent blockade of motor fibers. Such involvement could compromise the technique’s motor-sparing intent, highlighting the need for a refined technique. Future studies should explore the functional consequences of inadvertently blocking the parent common fibular nerve during a LAN block and aim to optimize the technique to minimize unintended nerve involvement, thereby ensuring both efficacy and safety in clinical applications.

Histological analysis confirmed the presence of articular nerves in most cases, supporting the accuracy of the gross dissection findings. However, the relatively low histological identification rate of the PAN (55%) is consistent with anatomical challenges encountered during dissection, reinforcing its role as a potential source of variability and reduced reproducibility in both research and clinical settings. These findings suggest that the PAN may be more difficult to consistently identify and target, potentially impacting the reliability of techniques aimed at its blockade. Future studies focusing on detailed anatomical mapping or advanced imaging of the PAN in diverse canine populations are warranted to improve the understanding of its trajectory and enhance the consistency of regional nerve blocks targeting this nerve. Despite the anatomical complexity of the popliteal fossa, the ultrasound-guided technique achieved a 100% success rate in staining the tissues between the popliteal artery and the caudal aspect of the femur as well as the external surface of the knee capsule where the PAN branches are located, highlighting the potential effectiveness of this technique in clinical cases. All the PAN injections were performed using a volume of injectate that was twice that of the MAN and LAN, based on the assumption that a larger surface area needed to be covered in the caudal quadrant of the knee capsule (PAN) compared to the more superficial and localized injection points for the MAN and LAN. Additionally, in a clinical setting, a volume of 0.1 mL kg^−1^ is easy to calculate and divide into four aliquots (one aliquot for the MAN, one for the LAN, and two for the PAN). However, the larger injectate volume remained localized in the proximal popliteal fossa (between the popliteal artery, the condyles of the femur, and the caudal external surface of the capsule); therefore, it is possible that a volume of 0.025 mL kg^−1^ may still be sufficient to desensitize the PAN. Further studies should aim to determine the minimal volume necessary to reliably desensitize the PAN, MAN, and LAN.

Incidents of intravascular and intraarticular dye contamination were observed with both PKD techniques, highlighting key safety considerations for their potential clinical application. Despite real-time needle visualization, the ultrasound-guided approach was not exempt from complications, with inadvertent arterial puncture, leading to minimal staining of the popliteal and the descending genicular arteries in 3 out of 20 injections. These findings underscore the importance of thorough image interpretation and suggest that the adjunctive use of Doppler imaging may enhance the identification of vascular structures and reduce the risk of vascular puncture. Although postmortem autolysis may have contributed to increased vascular permeability and passive dye diffusion into the vessels [28], intravascular contamination emphasizes the need for meticulous needle control during this procedure in live animals. Intra-articular staining was exclusively associated with the blind PAN approach, reinforcing the advantage of ultrasound guidance for accurate needle placement, particularly when targeting deep or anatomically crowded regions such as the popliteal fossa. These observations highlight the importance of operator experience and the potential for ultrasound to mitigate complications associated with blind injections in stifle analgesia.

Several limitations should be considered when interpreting the results of this study. The investigation was conducted on canine cadavers, where anatomical structures such as vessels, muscles, and ligaments may appear differently on ultrasonography compared to live animals. Therefore, the application and accuracy of the PKD technique in clinical settings may be affected. No comparison was made regarding the morphometric aspects of the knee joint and the relationship with neighboring structures across different dog breeds, ages, or sizes. Consequently, we cannot exclude that various morphometric aspects could impact the distribution of the injectate, particularly after PAN injection. Additionally, the spread of the injectate in cadavers may not mimic its behavior in living animals. In several instances during PAN injections, the target nerves could not be definitively located through gross anatomical evaluation. This resulted in defining successful injection as staining the area presumed to contain the articular branch, rather than direct nerve staining. Compounding this challenge, histological analysis failed to identify nerves in some specimens, even after multiple-step sections of the paraffin blocks. While this could stem from the absence of the target nerve within the submitted tissue or failure to capture these small nerves during sectioning, it highlights the anatomical variability and technical difficulty associated with the PAN. The difficulty in visualizing the PAN was previously described in the study by O’Connor and Woodbury [14], who reported the visualization of these articular branches in only 8 out of 18 specimens studied. These combined challenges underscore the need for further refinement of the techniques and methodologies (including dissection and histological processing) and injecting volumes to improve the consistency and accuracy of identifying and targeting articular nerves, particularly the PAN.

## 5. Conclusions

This study demonstrated the feasibility of PKD injection techniques for targeting the sensory branches of the knee joint capsule in dogs. The ultrasound-guided PKD technique appeared to be more reliable than the blind technique. By targeting the MAN, PAN, and LAN branches of the saphenous, tibial, and common fibular nerves, respectively, the PKD technique may provide effective analgesia of the knee and potentially enhance the quality of life in dogs affected by knee osteoarthritis or provide pain relief following knee surgery. Further studies are necessary to assess the clinical efficacy and safety of this technique in providing stifle analgesia in canine patients.

## Data Availability

The raw data supporting the conclusions of this article will be made available by the authors upon request.
